# Mesenchymal Stem Cell-Conditioned Medium Induces Neutrophil Apoptosis Associated with Inhibition of the NF-κB Pathway in Endotoxin-Induced Acute Lung Injury

**DOI:** 10.3390/ijms20092208

**Published:** 2019-05-05

**Authors:** Vincent Yi-Fong Su, Chi-Shiuan Lin, Shih-Chieh Hung, Kuang-Yao Yang

**Affiliations:** 1 Department of Internal Medicine, Taipei City Hospital, Yangming Branch, Taipei 11146, Taiwan; bsbipoke@hotmail.com; 2 Institute of Clinical Medicine, Yang-Ming University, Taipei 11221, Taiwan; 3 School of Medicine, National Yang-Ming University, Taipei 11221, Taiwan; 4 School of Chinese Medicine for Post-Baccalaureate of I-Shou University, Kaohsiung 82445, Taiwan; chishiuanlin0520@gmail.com; 5 Center for Traditional Medicine Taipei Veterans General Hospital, Taipei 11217, Taiwan; 6 Department of Orthopaedics and Traumatology Taipei Veterans General Hospital, Taipei 11217, Taiwan; hung3340@gmail.com; 7 Therapeutical and Research Center of Musculoskeletal Tumor Taipei Veterans General Hospital, Taipei 11217, Taiwan; 8 Institute of Biomedical Sciences, Academia Sinica, Taipei 11529, Taiwan; 9 Integrative Stem Cell Center, China Medical University, Taichung 40402, Taiwan; 10 Graduate Institute of Clinical Medical Science, China Medical University, Taichung 40402, Taiwan; 11 Institute of Emergency and Critical Care Medicine Yang-Ming University, Taipei 11221, Taiwan; 12 Department of Chest Medicine; Taipei Veterans General Hospital, Taipei 11217, Taiwan

**Keywords:** mesenchymal stem cell, neutrophil apoptosis, acute lung injury

## Abstract

The immunomodulatory effects of mesenchymal stem cells (MSCs) are established. However, the effects of MSCs on neutrophil survival in acute lung injury (ALI) remain unclear. The goal of this study was to investigate the effect of an MSC-conditioned medium (MSC-CM) on neutrophil apoptosis in endotoxin-induced ALI. In this study, an MSC-CM was delivered via tail vein injection to wild-type male C57BL/6 mice 4 h after an intratracheal injection of lipopolysaccharide (LPS). Twenty-four hours later, bronchoalveolar lavage fluid (BALF) and lung tissue were collected to perform histology, immunohistochemistry, apoptosis assay of neutrophil, enzyme-linked immunosorbent assays, and an electrophoretic mobility shift assay. Human neutrophils were also collected from patients with sepsis-induced acute respiratory distress syndrome (ARDS). Human neutrophils were treated in vitro with LPS, with or without subsequent MSC-CM co-treatment, and were then analyzed. Administration of the MSC-CM resulted in a significant attenuation of histopathological changes, the levels of interleukin-6 and macrophage inflammatory protein 2, and neutrophil accumulation in mouse lung tissues of LPS-induced ALI. Additionally, MSC-CM therapy enhanced the apoptosis of BALF neutrophils and reduced the expression of the anti-apoptotic molecules, Bcl-xL and Mcl-1, both in vivo and in vitro experiments. Furthermore, phosphorylated and total levels of nuclear factor (NF)-κB p65 were reduced in lung tissues from LPS + MSC-CM mice. Human MSC-CM also reduced the activity levels of NF-κB and matrix metalloproteinase-9 in the human neutrophils from ARDS patients. Thus, the results of this study suggest that the MSC-CM attenuated LPS-induced ALI by inducing neutrophil apoptosis, associated with inhibition of the NF-κB pathway.

## 1. Introduction

Acute lung injury (ALI) is an inflammatory disease which can lead to the development of acute respiratory distress syndrome (ARDS) and significant morbidity and mortality [1]. Neutrophils are the major inflammatory cells that are recruited to inflamed lungs and they play a central role in the pathogenesis of ALI. Pulmonary edema and endothelial and epithelial cell injury are also observed in cases of ALI [2]. Lipopolysaccharide (LPS) has been shown to cause excessive activation and/or delayed apoptosis in neutrophils, thereby contributing to tissue damage [3]. It is hypothesized that neutrophil apoptosis is needed for the resolution of ARDS and restoration of tissue homeostasis [4,5]. Nuclear factor (NF)-κB is a transcription factor that also regulates the expression of proinflammatory and anti-apoptotic genes during the development of ALI and ARDS [6].

The potential for mesenchymal stem cells (MSCs), including hematopoietic stem/progenitor cells (HSPCs) and induced pluripotent cells (iPSCs), to contribute to tissue repair in ALI has been reported [7]. For example, previous studies of experimental mouse models have shown that MSCs can attenuate endotoxin-induced ALI and improve survival [8,9] by mediating broad immunomodulatory effects [10]. Accumulating evidence further supports a role for soluble factors. To date, the exact mechanism underlying the therapeutic effects of MSCs remains unclear, although MSCs are widely used in clinical stem cell therapy due to their low immunogenicity and accessibility for collection.

In principle, MSCs and iPSCs mediate different and potent immunomodulatory effects [11]. We previously demonstrated that the intravenous delivery of iPSCs can attenuate the severity of endotoxin-induced ALI by enhancing GRK2 activity and reducing the expression of TREM-1 to regulate neutrophil chemotaxis in inflamed lungs [12,13,14]. In the present study, the effects of an MSC-conditioned medium (MSC-CM) on neutrophil apoptosis due to LPS-induced ALI were investigated.

## 2. Results

### 2.1. Effects of MSC-CM on the Histopathology of Endotoxin-Induced ALI

**Figure 1:**
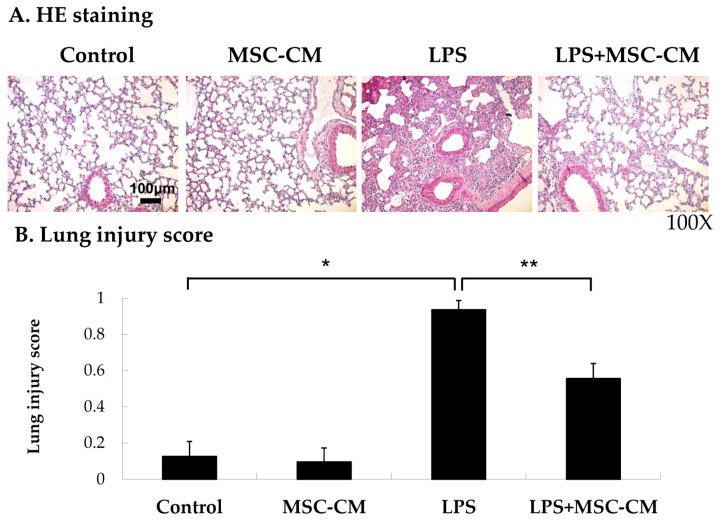
A tail vein injection of the MSC-CM improved the histological features of LPS-induced ALI in male C57BL/6 mice. (**A**) H&E staining demonstrates that lung injury was significantly attenuated in the mice that received LPS + MSC-CM compared with LPS alone. The control mice and MSC alone mice exhibited minimal histopathologic abnormalities. Magnification = 100×. Scale bar = 100 μm. (**B**) The lung injury scores were also significantly decreased in the mice that received LPS + MSC-CM compared with LPS alone. Data are presented as the mean ± SD. * *p* < 0.05, compared to the controls; ** *p* < 0.05, compared to the LPS group; *n* = 6 per group.

Intratracheal injections of LPS into C57BL/6 mice resulted in extensive ALI after 4 h. The induced lung injury was confirmed histopathologically and characterized by lung edema, alveolar spaces filled with mononuclear/neutrophilic infiltrates, and thickening of the alveolar walls and interstitium (Figure 1A). When the induction of ALI was followed by a tail vein injection of the MSC-CM 4 h later, histological evaluations showed that the numbers of infiltrative neutrophils and injured areas were significantly reduced after 24 h compared to the lung tissues with ALI that did not receive MSC-CM treatment (the LPS group) (Figure 1A). Lung injury scores for the control, MSC-CM, LPS, and LPS + MSC-CM groups were consistent with these results (Figure 1B). There were no significant differences in the scores between the mice treated with the MSC-CM alone and the control mice, while the LPS group had a significantly higher lung injury score compared with the control group (Figure 1B). When the MSC-CM was administered following LPS, the lung injury score was significantly reduced compared with that of the LPS group.

### 2.2. Effects of MSC-CM on Proinflammatory Cytokines in Lung Tissues

**Figure 2:**
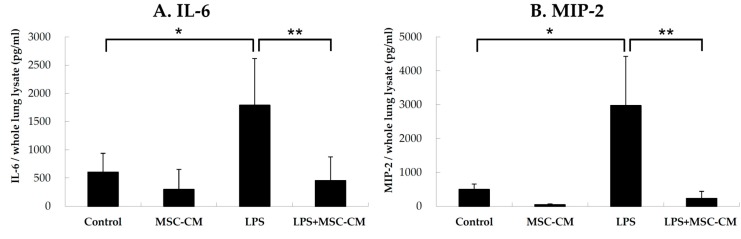
Administration of the MSC-CM decreased the levels of the proinflammatory cytokines, IL-6 and MIP-2, in BALF collected from mice with ALI. In ELISA assays, significantly higher concentrations of (**A**) IL-6 and (**B**) MIP-2 were detected in BALF samples after LPS exposure (LPS) compared to the control group. In contrast, administration of the MSC-CM after LPS (LPS+MSC-CM) resulted in a significant reduction in IL-6 and MIP-2 levels compared to the LPS group. Data are presented as the mean ± SD. * *p* < 0.05, compared to the controls; ** *p* < 0.05, compared to the LPS group; *n* = 6 per group.

IL-6 and MIP-2 are well-characterized proinflammatory markers due to their increased expression in a number of inflammatory diseases, including ALI. The expression of IL-6 was detected in lung tissue extracts prepared from control, MSC-CM, LPS, and LPS + MSC-CM mouse lungs. ELISA data showed that significantly higher concentrations of IL-6 were present in the LPS group compared to the control group (Figure 2A). Similarly, when the expression of MIP-2 was detected in lung extracts collected from the four experimental mouse groups (with MIP-2 also being a marker of neutrophil recruitment), the highest concentration of MIP-2 was in the LPS group compared to the control (Figure 2B). For both IL-6 and MIP-2, their levels were significantly reduced following administration of the MSC-CM (LPS+MSC-CM) compared to the LPS group (Figure 2). However, there were no significant differences in the levels of IL-6 and MIP-2 between the mice treated with the MSC-CM alone (MSC) and the control mice (Control).

### 2.3. Effects of MSC-CM on Neutrophil Accumulation in Lung Tissues

**Figure 3:**
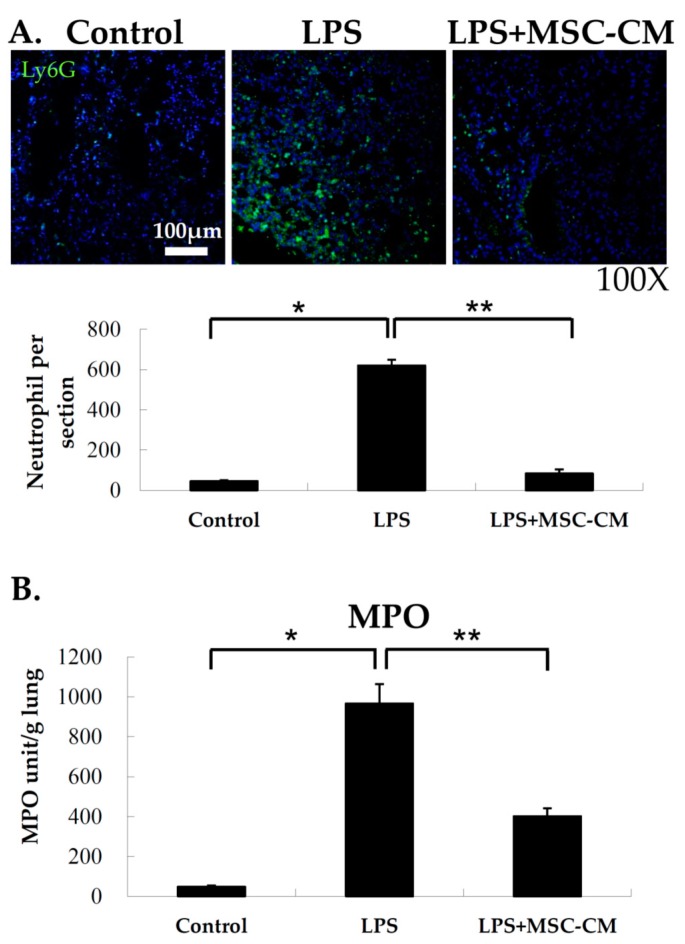
MSC-CM reduced neutrophil accumulation in the lungs of LPS-induced ALI mice. (**A**) Neutrophil accumulation was examined with immunofluorescent staining of lung tissues for Ly6G, a neutrophil marker. Neutrophil accumulation in alveoli significantly increased after LPS exposure (LPS) and was significantly reduced following administration of the MSC-CM (LPS+MSC-CM). Green: Ly6G (lymphocyte antigen 6G); Blue: 4’,6-diamidino-2-phenylindole (DAPI). Magnification = 100×. Scale bar = 100 μm. The number of neutrophils per section for lung tissue samples stained for Ly6G are presented as the mean ± SD. (**B**) Myeloperoxidase (MPO) activity was detected in whole lung tissue lysates and the data indicate that there was significant neutrophil accumulation. LPS mice (LPS) had a higher level of MPO in the lung compared to the control mice, whereas the mice that received LPS + MSC-CM had decreased levels of MPO compared to LPS mice. Data are presented as the mean ± SD. * *p* < 0.05, compared to the controls; ** *p* < 0.05, compared to the LPS group; *n* = 6 per group.

Following administration of the MSC-CM after LPS-induced ALI, neutrophil accumulation in the lungs of the various experimental groups were examined based on staining of the neutrophil marker, Ly6G. As shown in Figure 3A, the signal for Ly6G staining was significantly greater in the LPS group compared to the control group. This result indicates that neutrophils greatly accumulated in the lungs of the mice that received LPS. However, following LPS with a tail vein injection of MSC-CM, a significant decrease in neutrophil accumulation was observed with a reduction in Ly6G staining (Figure 3A). To confirm these results and to quantify neutrophil accumulation in the lung, MPO activity was assayed for the three experimental groups. MPO activity was higher in the lung tissue extracts collected from the LPS group compared with the control group, and this activity level was reduced following the treatment of the MSC-CM (Figure 3B). Taken together, these results demonstrate that neutrophil accumulation in response to LPS-induced ALI was attenuated following the injection of the MSC-CM.

### 2.4. Effects of MSC-CM on Neutrophil Apoptosis in ALI

**Figure 4:**
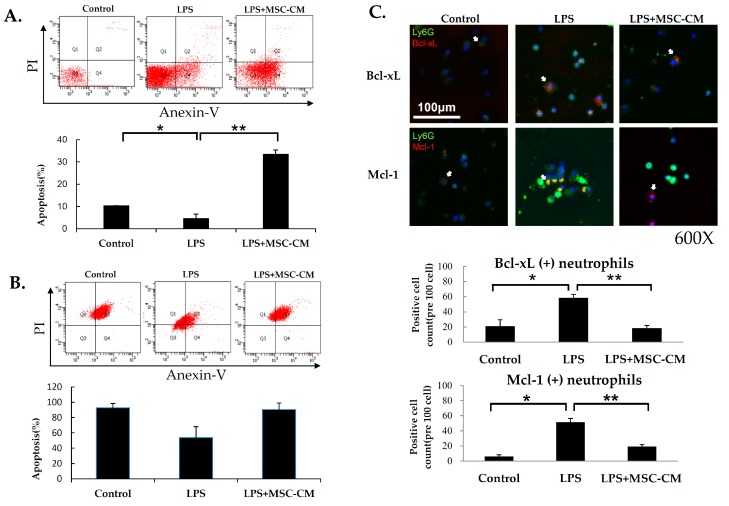
Apoptosis of pulmonary neutrophils were significantly induced by MSC-CM treatment in vivo and vitro models. (**A**) In vivo model, the ratio of apoptotic neutrophils present in BALF samples collected from the mice with LPS-induced ALI (LPS) (4.6 ± 2%) contained a lower ratio of apoptotic neutrophils compared with control mice (Control) (10.3 ± 0.15%). In contrast, the ratio of apoptotic neutrophils was higher in the mice that received LPS + MSC-CM (LPS+MSC-CM) (33.4 ± 2%) than LPS alone (LPS). The bar graph below presents the percentage apoptosis for each experimental group. (**B**) In vitro model, the neutrophils isolated from BALF samples collected from the mice with LPS-induced ALI and cultured 2 h with or without LPS and MSC-CM treatment in vitro. Mouse neutrophils with LPS in vitro stimulation (LPS) (53.7 ± 14.3%) contained a lower ratio of apoptosis compared with control neutrophils (Control) (92.7 ± 5.5%). In contrast, the ratio of apoptotic neutrophils was higher in neutrophils that received LPS + MSC-CM (90.4 ± 8.6%) compared with neutrophils receiving LPS only (LPS). The bar graph below presents the percentage apoptosis for each experimental group. (**C**) Expression of Bcl-xL and Mcl-1 in pulmonary neutrophils of BALF samples increased in the mice with LPS-induced ALI (LPS) compared to the control mice (Control), while treatment with MSC-CM(LPS+MSC-CM)markedly reduced both the expression of Bcl-xL and Mcl-1. Data are presented as the mean ± SD. PI: propidium iodine; Blue: DAPI (4’,6-diamidino- 2-phenylindole); Green (FITC): Ly6G (lymphocyte antigen 6G); Red (Cy5): Bcl-xL or Mcl-1. Magnification = 400×. Scale bar = 100 μm. Arrows indicate positive cells. Immunofluoresence intensity was quantitated for the control, LPS, and LPS-MSC tissue sections for the staining of Bcl-xL and Mcl-1 and the data are presented as the mean ± SD. * *p* < 0.05, compared to the controls; ** *p* < 0.05, compared to the LPS group; *n* = 6 per group.

Neutrophil apoptosis was assayed following the administration of LPS with and without the subsequent MSC-CM treatment. To detect neutrophil apoptosis, BALF were prepared, stained with Annexin V-FITC, and analyzed by flow cytometry (Figure 4A). A significant decrease in the ratio of apoptotic neutrophils from 10.3% in the control group to 4.6% in the LPS-stimulated group was detected. These results indicate that LPS was able to significantly prolong the survival of neutrophils in the lungs of our experimental model. In contrast, administration of the MSC-CM after LPS resulted in a significant induction of neutrophil apoptosis (33.4%). To confirm these findings with the in vitro model, mouse neutrophils purified from BALF samples were collected from the mice with LPS-induced ALI and cultured for 2 h with or without LPS and MSC-CM treatment in vitro. Neutrophils with LPS in vitro stimulation (LPS) (53.7%) had less apoptosis compared with control neutrophils (Control) (92.7%). In contrast, neutrophils that received LPS + MSC-CM (90.4%) treatment had more apoptosis compared with neutrophils receiving LPS only (LPS) (Figure 4B). To explore these results, neutrophils present in BALF samples collected from control, LPS, and LPS + MSC-CM mice were stained for Bcl-xL and Mcl-1, which represent anti-apoptotic proteins with roles in the intrinsic apoptosis pathway. Stronger immunofluorescence staining was observed for both Bcl-xL and Mcl-1 in the neutrophils of BALF from LPS mice (Figure 4C) compared with neutrophils from control mice. In contrast, the administration of LPS followed by MSC-CM treatment resulted in a markedly lower staining signal for Bcl-xL and Mcl-1 in the BALF neutrophils compared with the administration of LPS alone.

### 2.5. Effects of MSC-CM on the Expression of NF-kB and MMP-9 in Mouse and Human Neutrophils

**Figure 5:**
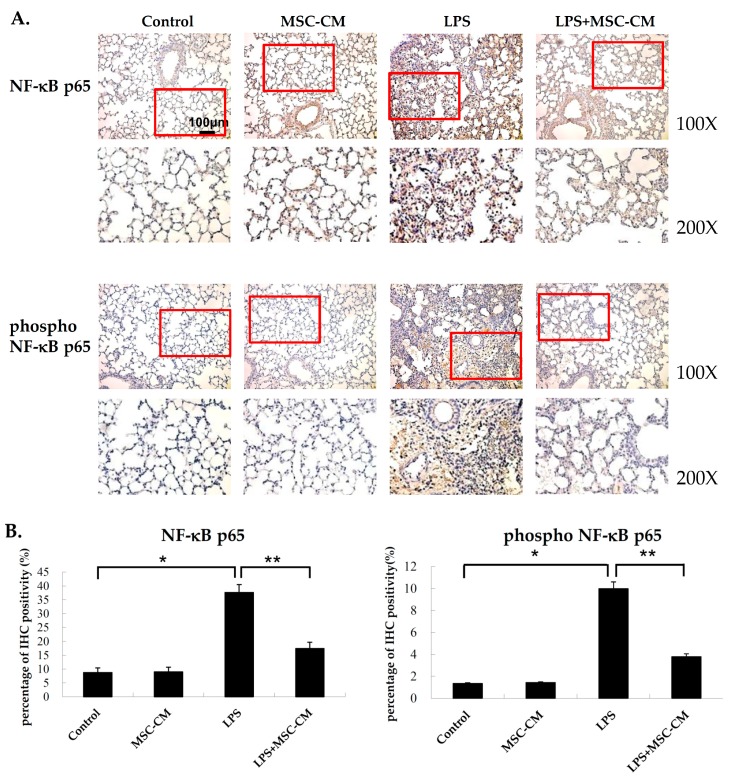
Administration of the MSC-CM reduced the expression levels of total and phosphorylated NF-kB p65 in mice with LPS-induced ALI. (**A**) Lung tissues from the mice challenged with LPS (LPS) exhibited more positive staining for total NF-kB p65 than control mice (Control). In contrast, lung tissues from mice that received LPS + MSC-CM exhibited a significant decrease in total NF-kB p65 staining compared to the LPS mice. Staining of phospho-NF-kB p65 was enhanced in lung tissues from the LPS mice compared with control mice, and staining was significantly reduced in lung tissues from the mice that received LPS + MSC-CM compared to the LPS mice. (**B**) The quantification of IHC staining showed the similar results. Magnification = 100× and 200× (red box). Scale bar = 100 μm. For both sets of staining, quantitation of staining intensity is presented as the mean ± SD in bar graphs. * *p* < 0.05, compared to the controls; ** *p* < 0.05, compared to the LPS group; *n* = 6 per group.

To detect the expression of the total and phosphoryated forms of NF-kB p65, IHC assays were performed. The lung tissue sections that were prepared from the mice with LPS-induced ALI exhibited stronger staining of both total and phospho-NF-kB compared to the control mice (Figure 5). In contrast, the lung tissues that were exposed to LPS and then MSC-CM treatment showed a marked reduction in staining intensity for total and phospho-NF-kB (Figure 5).

**Figure 6:**
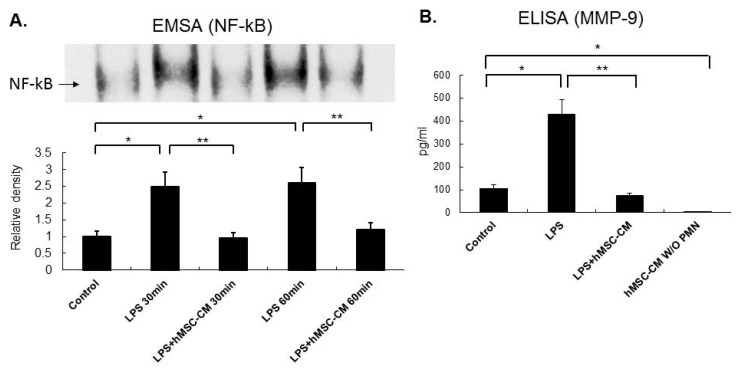
The human MSC-CM (hMSC-CM) reduced NF-kB activation and the expression of MMP-9 in human neutrophils isolated from patients with ARDS. (**A**) Human neutrophils that were treated with LPS for 30 min or 60 min in vitro exhibited a significant increase in NF-kB activity in EMSA analyses compared to the control neutrophils. In contrast, human neutrophils that underwent LPS treatment (30 min or 60 min) and hMSC-CM (LPS+hMSC-CM) showed a marked reduction in NF-kB activation compared to LPS-treated neutrophils. (**B**) In ELISA assays of MMP-9 activity from culture mediums of human neutrophils in vitro, greater activity was observed after LPS treatment, while a marked reduction in MMP-9 activity was observed after LPS + hMSC-CM treatment. Data are presented as the mean ± SD. * *p* < 0.05, compared to the controls; ** *p* < 0.05, compared to the LPS group; *n* = 6 per group.

Human neutrophils were also isolated from peripheral blood samples collected from patients with sepsis-induced ARDS. These neutrophils were incubated with LPS for 30 min or 60 min in vitro and were then incubated with or without human MSC condition medium (hMSC-CM). A significant increase in NF-kB activity was detected in the EMSAs performed for human neutrophil with the 30 min and 60 min LPS treated in vitro compared to the untreated human neutrophils (Figure 6A). Consistent with the mouse neutrophil results, human neutrophils that underwent LPS treatment, followed by exposure to hMSC-CM, showed a marked reduction in the expression of NF-kB. When MMP-9 activity was assayed for the culture medium from human neutrophil that were untreated, treated with LPS 60 min, or treated with LPS followed by hMSC-CM 60 min in vitro, similar findings were obtained with a marked increase in MMP-9 activity observed on LPS stimulation, and a significant decrease in MMP-9 activity observed on LPS stimulation followed by hMSC-CM treatment (Figure 6B).

## 3. Discussion

There are four major findings in the present study regarding administration of the MSC-CM following LPS-induced ALI: (i) the number and activity of neutrophils in the lungs were reduced; (ii) levels of IL-6, MIP-2, NF-κB p65, and phospho-NF-κB p65 were reduced; (iii) expression levels of Bcl-xL and Mcl-1 were reduced and neutrophil apoptosis was affected; and (iv) lower levels of NF-kB and MMP-9 activity were detected in human neutrophils that were isolated from the peripheral blood of patients with ARDS and treated with LPS + hMSC-CM. Thus, to our knowledge, this is the first study to report that the MSC-CM can regulate neutrophil apoptosis in ALI.

During the early phase of ALI, neutrophils are one of the first immune cells to respond and migrate towards the site of inflammation. Consequently, neutrophils represent the first line of innate immunity and they are an essential component in the pathogenesis of ALI. Neutrophils are remarkably short-lived, with a half-life of 6–8 h [15]. However, when neutrophils are excessively activated and their apoptosis is delayed, lung tissue damage can occur [16]. It has been hypothesized that restoration of neutrophil apoptosis is required for the resolution of inflammation in the lung. In previous studies, it has been demonstrated that the population of apoptotic neutrophils in the lungs of patients with ARDS is very small [17]. Moreover, neutrophil apoptosis has been found to be inversely proportional to the severity of sepsis-induced ARDS [16]. For infected tissues, the presence of microbial factors such as LPS and pro-inflammatory stimuli can induce a delay in neutrophil apoptosis [18,19] and the inhibition of neutrophil apoptosis may contribute to the development of ALI [20]. Conversely, amelioration of ALI may involve an enhancement of neutrophil apoptosis [21]. In the current study, the MSC-CM was able to attenuate LPS-induced ALI by enhancing apoptosis of neutrophils. Thus, for the treatment of ALI induced by LPS, the ability to regulate apoptosis in neutrophils represents an important target in attenuating or resolving LPS-induced inflammation.

NF-κB induces the transcription of various proinflammatory cytokines and it plays a central role in the development of ALI [22]. Accordingly, the endotoxin-induced activation of NF-κB in neutrophils has also been shown to play a major role in the development of ALI [23]. More recently, it has been demonstrated that apoptosis of human neutrophils is regulated by NF-κB [24,25], with inhibition of NF-κB promoting neutrophil apoptosis and reducing lung damage [23,26,27]. We previously reported that alterations in the early stages of neutrophil activation affect the clinical outcome of ALI [14]. For example, patients with neutrophils that did not exhibit an increase in nuclear translocation of NF-κB after exposure to LPS had a significantly shorter time on a ventilator than patients with neutrophils that did exhibit NF-κB activation [14]. Thus, regulation of the NF-kB signaling pathway in neutrophils may represent a therapeutic target for the treatment of inflammatory diseases such as ALI.

MSCs have been shown to mediate potent immunomodulatory effects to influence both innate and adaptive immune cells. Thus, the possible benefits of MSC-based treatments for ALI have been investigated [28]. We previously reported that iPSCs can be used to reduce the severity of endotoxin-induced ALI and to effectively regulate neutrophil chemotaxis in lungs [12,13]. In this study, administration of the MSC-CM was able to attenuate the levels of IL-6 and MIP-2 in mouse lung tissues of LPS-induced ALI. The murine chemokine MIP-2 is one of the major chemoattractants responsible for neutrophil recruitment [29,30]. Meanwhile, Raffaghello et al. [31] reported that MSCs can prevent neutrophil apoptosis and degranulation in cultures in the absence of cell–cell contacts, and IL-6 may be a key MSC-derived soluble factor in prolonging the survival of neutrophils in the bone marrow niche. In the present study, we provided the first evidence that stem cell-conditioned medium can inhibit the NF-kB pathway to induce neutrophil apoptosis in endotoxin-induced ALI. In addition, we demonstrated that intravenous administration of the MSC-CM significantly diminished histopathological changes associated with LPS-induced ALI, and this involved a significant reduction in the number and activity of neutrophils in lung tissue as confirmed by the immunostaining of Ly6G and assays of MPO activity. Moreover, similar effects were observed for the human MSC-CM on neutrophils isolated from patients with ARDS, with reduced NF-kB and MMP-9 activities characterizing these neutrophils following LPS + MSC-CM treatment. Taken together, these findings indicate that MSCs mediate more complex effects on neutrophils than their classical role as immune suppressor cells would suggest. Additionally, neutrophils were described as releasing their nuclear contents into the extracellular spaces in acute response to systemic inflammation or infection, termed neutrophil extracellular trap (NET) formation. Recently, NETs have been shown to be potential mediators in LPS-induced ALI [32] and the excessive presence of NETs may result in adverse effects in ALI. In summary, targeting NETs may represent a potentially effective method to treat ALI. However, to our knowledge, there have been no studies investigating the effects of MSCs on NET formation in ALI. Consequently, neutrophils may represent one of the most important immune cell types in regulating the immunomodulatory effects of stem cell therapy in ALI.

## 4. Materials and Methods

All animal experiments were conducted in accordance with institutional animal care and use committee (IACUC)-approved protocols. In addition, all experiments were approved by the Institutional Review Board of Taipei Veterans General Hospital (VGHIRB No. 201012033IC, 08/02/2011). Informed consent was obtained from all participating patients or their surrogates prior to enrollment.

### 4.1. Mice

Male C57BL/6 mice (8–12 weeks old, weighing 20–25 g) were purchased from the National Experimental Animal Center (Taipei, Taiwan). The animals were kept in standard plastic animal cages with husk bedding at 25  ± 2 °C with a 12-h light/dark cycle. All of the mice were maintained under specific-pathogen-free conditions and food and water were provided ad libitum.

### 4.2. Experimental Design

A mouse model of endotoxin-induced ALI was established as previously described [12,13,14], with some modifications. Briefly, mice were anesthetized and then administered an intratracheal injection of LPS (Escherichia coli 0111:B4 endotoxin; Sigma, St. Louis, MO, USA) at a dose of 5 mg/kg in 50 µL phosphate-buffered saline (PBS). A similar dose produced acute neutrophilic alveolitis in mice, which is histologically consistent with ALI in mice [12,13,14]. As a control, mice received an intratracheal injection of 50 µL PBS. Four hours later, the two groups of mice received either 200 µL MSC-CM or 200 µL PBS via tail vein injection to establish an MSC-CM-treated ALI group and a PBS-treated ALI group, respectively. This MSC-CM load has previously been used in our laboratory and has achieved a rescue effect from other injuries. Twenty-four hours after the tail vein injections, lung tissues were collected from each mouse to assess lung injury and histology. These tissues were also subjected to immunohistochemistry (IHC), flow cytometry, immunofluorescence, and bronchoalveolar lavage fluid (BALF) assays. Detailed information on the in vitro study is presented in the supplementary section.

### 4.3. Generation of Murine and Human MSC-CM

Mouse MSCs were obtained from C57BL/6 mice and expanded at the Stem Cell Research Center, Taipei Veterans General Hospital (Taipei, Taiwan), as described previously [33]. MSCs were seeded at 10,000 cells per cm^2^ and incubated in a complete culture medium for 1 day. The attached cells were washed three times with PBS, and the medium was replaced with serum-free basal medium and incubated for 48 h. The MSC-CM was collected, centrifuged at 1500× *g* for 10 min to remove cell debris, and further concentrated 50 × by ultrafiltration using centrifugal filter units with a 5 kDa cut-off (Millipore, Billerica, MA, USA) following the manufacturer’s instructions. Human MSCs were also acquired from the Stem Cell Research Center (Taipei Veterans General Hospital, Taipei, Taiwan) and used to generate a human mesenchymal stem cell-conditioned medium (hMSC-CM). All of these procedures were conducted in our collaborator’s laboratory under the direction of Dr. Hung.

### 4.4. Isolation of Neutrophils from BALF

Detailed information is presented in the supplementary section.

### 4.5. Histology and IHC of Lung Tissue Sections

Detailed information is presented in the supplementary section.

### 4.6. Lung Injury Scoring of Lung Tissue Sections

Detailed information is presented in the supplementary section.

### 4.7. Enzyme-Linked Immunosorbent Assay (ELISA) Assay

Detailed information is presented in the supplementary section.

### 4.8. Confocal Microscopy

Detailed information is presented in the supplementary section.

### 4.9. Myeloperoxidase (MPO) Activity Assay

Detailed information is presented in the supplementary section.

### 4.10. Flow Cytometry

Detailed information is presented in the supplementary section.

### 4.11. Patients with ARDS and Isolation of Human Neutrophils

Patients with sepsis-induced ARDS were eligible for inclusion in this study according to the inclusion criteria detailed in our previous study [34]. Neutrophils were obtained from peripheral blood samples that were collected from these patients according to the details provided in the supplementary section.

### 4.12. Electrophoretic Mobility Shift Assay (EMSA)

Detailed information is presented in the supplementary section.

### 4.13. Apoptosis Assay

For the in vitro apoptosis assay, BALF from LPS-induced mice were collected as per the details in the supplementary section and the Annexin V apoptosis kit (BD, 556547, San Jose, CA, USA) was used for cell staining, which followed the kit’s protocol.

### 4.14. Statistical Analysis

Detailed information is presented in the supplementary section.

## 5. Conclusions

In this study, MSC-CM attenuating endotoxin-induced ALI was identified in relation to the increase in neutrophil apoptosis. These findings were associated with the decreases in the expression of anti-apoptotic molecules, Bcl-xL and Mcl-1, and the NF-κB pathway in neutrophils.

## Data Availability

The datasets used and/or analysed during the present study are available from the corresponding author on reasonable request.

## Supplementary Materials

Supplementary materials can be found at https://www.mdpi.com/1422-0067/20/9/2208/s1.
