# Supplementary material for: Mesenchymal Stem Cell-Conditioned Medium Induces Neutrophil Apoptosis Associated with Inhibition of the NF-κB Pathway in Endotoxin-Induced Acute Lung Injury
Source: Int J Mol Sci. 2019 May 5;20(9):2208. doi: 10.3390/ijms20092208 (PMC6540353; doi:10.3390/ijms20092208)
Supplement: Supplementary file 1 [file ijms-20-02208-s001.pdf]

Mesenchymal Stem Cell-Conditioned Medium Induces Neutrophil Apoptosis Associated with Inhibition of the NF-κB Pathway in Endotoxin-Induced Acute Lung Injury

MATERIALS AND METHODS

Histology and IHC of Lung Tissue Sections

Lungs from each group were excised 24 h after endotoxin-induced ALI. HE and IHC staining was performed on 4-μm paraffin sections of formalin-fixed lung samples. The samples were deparaffinized and rehydrated. After blocking endogenous peroxidase with 0.3% H_2O_2 for 30 min at room temperature, NF-κB p65 (sc-8008, Santa Cruz Biotechnology, Dallas, USA) and phospho-NF-κB p65 (#3033, Cell Signaling Technologies, Danvers, MA) were added, and the mixture left at 4 °C overnight. The sections were then incubated with peroxidase-conjugated Envision® + Dual Link System-HRP (DAB+) kits (K4065, DAKO, Carpinteria, CA) for 1 h at room temperature and developed with diaminobenzidine chromogen substrate (K4065 kit). After immunohistochemical staining, the sections were incubated with eosin for 1 min and then dehydrated. Electronic images of the immunohistochemical and HE staining were taken with an Olympus AX80 fluorescence microscope (Olympus America, Melville, NY). Scoring of histological sections was done according to the recommendation of the American Thoracic Society. To quantitate the percentage of cells that were positive for total NF-κB p65 or phospho-NF-κB p65, ten 200x magnification fields were randomly selected and the cells counted. To quantitatively analyze the IHC intensity of the scratched area, the percentage of IHC signaling per photographed field was analyzed using Image processing software (Image-Pro Plus, Media Cybernetics Inc., Silver Spring, MD).

Lung Injury Scoring of Lung Tissue Sections

To quantify the severity of ALI by histology, the lung injury score was assessed. Two investigators independently evaluated each hematoxylin and eosin (H&E)-stained slide in a blinded manner. To generate the lung injury score, 300 alveoli were counted on each slide at 400× magnification. Within each field, points were assigned according to the predetermined criteria used in a previous study.(1) Lung injury score = [(alveolar hemorrhage points/no. of fields) + 2 × (alveolar infiltrate points/no. of fields) + 3 × (fibrin points/no. of fields) + (alveolar septal congestion/no. of fields)]/total number of alveoli counted.

ELISA Assay

After the lung vascular beds had been flushed by injecting 5 mL of chilled (4 °C) PBS into the right ventricle, the lungs were collected and then homogenized for 30 s in a lysis buffer containing 10 mM HEPES, 150 mM NaCl, 1 mM EDTA, 0.6% Ipegal, 5 mM PMSF, 1 μg/mL leupeptin, 1 μg/mL aprotinin, 10 μg/mL soybean trypsin inhibitor, and 1 μg/mL pepstatin. The homogenates were centrifuged at 20,000× *g* at 4 °C for 10 min and the supernatants were collected. The protein content of the supernatants was determined using a bicinchoninic acid protein assay kit (Pierce, Rockford, IL). Immunoreactive IL-6 and MIP-2 were quantitated using commercially available ELISA kits (IL-6: M6000B, MIP-2： MM200, R&D Systems, Minneapolis, MN), The minimum detectable dose of mouse MIP-2 and IL-6 was 1.5 pg/mL and 1.3–1.8 pg/mL, respectively. Supernatants from isolated human neutrophil experiments were used to detect MMP-9 activity with an Amersham Matrix Metalloproteinase-9 (MMP-9) Biotrak Activity Assay System (RPN2634, GE Health Care Life Science, Buckinghamshire, UK). The threshold of sensitivity for MMP-9 was 0.5 ng/mL.

Myeloperoxidase (MPO) Activity Assay

At the end 24 h, the excised lungs from three to four mice per treatment group were frozen in liquid nitrogen, weighed, and stored at −80 °C. The lungs were homogenized for 30 s in 1.5 mL 20 mM potassium phosphate, pH 7.4, and centrifuged at 4 °C for 30 min at 26,000× *g*. The pellets were resuspended in 1.5 mL 50 mM potassium phosphate, pH 6.0, containing 0.5% hexadecyltrimethylammonium bromide, sonicated for 90 s, incubated at 60 °C for 2 h, and then centrifuged. The supernatant was assayed for peroxidase activity corrected to lung weight.

Isolation of Neutrophils from BALF

The mice were anesthetized, and their tracheae were cannulated using a catheter. PBS (0.5 mL) was infused three times into the lungs to collect BALF. Cells in the BALF were counted with a hemocytometer and prepared for flow cytometry and confocal microscopy.

Flow Cytometry

Pulmonary neutrophils isolated from BALF were prepared for in vivo and in vitro apoptosis assay. In vivo model, neutrophils were directly stained with annexin-V/propidium iodide and measured by flow cytometry. In vitro model, neutrophils purified from the BALF samples of mice with LPS-induced ALI were cultured with or without 100 ng/mL LPS. MSC-CM was added to the neutrophil cultures for 2 hours with LPS stimulation, and then all measured by flow cytometry. Apoptotic cells were detected and quantified using the annexin-V/propidium iodide method with a TACS Annexin-V-FITC kit (TA4638/TA5532, R&D Systems, Minneapolis, MN, USA) according to the manufacturer's instructions. Binding of fluorescein isothiocyanate-labeled annexin-V and propidium iodide staining of the cells were determined by flow cytometry on a FACSCalibur system (BD, State of New Jersey, USA).

Confocal Microscopy

Confocal microscopy was performed to detect the expressions of Bcl-xL and Mcl-1. Cells in the BALF were subjected to cytospinning, fixed, permeabilized, and stained with Bcl-xL(sc-7195, Santa Cruz Biotechnology, Dallas, USA) and Mcl-1(sc-819, Santa Cruz Biotechnology, Dallas, USA) antibodies and the corresponding Cy5-labeled secondary antibodies (ab6564, abcam, Cambridge, MA, USA). Stained cells were counterstained with DAPI and analyzed with an Olympus FV10i confocal microscope.

Patients with ARDS and Isolation of Human Neutrophils

Patients with sepsis-induced ARDS were eligible for inclusion in this study according to the inclusion criteria detailed in our previous study.(2) Neutrophils in peripheral blood were obtained from the patients with sepsis-induced ARDS. All experiments were approved by the Institutional Review Board of Taipei Veterans General Hospital (VGHIRB No. 201012033IC). Consent was obtained from all patients or their surrogates before enrollment.

Peripheral blood was obtained from the patients, and neutrophils (purity > 98%) were isolated by plasma-Percoll gradients after dextran sedimentation of erythrocytes. The neutrophils were resuspended at a final concentration of 5×10^6 cells/mL in RPMI1640 containing 5% fetal calf serum and cultured with or without 100 ng/mL LPS. hMSC-CM (20%) was added to the neutrophil cultures for 30-60 min with LPS stimulation at the same time. The supernatants of isolated human neutrophil cultures with or without 100 ng/ml LPS stimulation for 1 h were used for the detection of MMP-9 activity.

Electrophoretic Mobility Shift Assay (EMSA)

Nuclear extracts were prepared as previously described.(2) Isolated human neutrophils from the patients with ARDS were incubated for 15 min in buffer A (10 mM HEPES [pH 7.9], 1.5 mM $MgCl_2$, 10 mM KCl, pH 7.9). After the cytoplasm had been removed from the nuclei by 15 passages through a 25-gauge needle, the nuclei were collected by centrifugation at 600× *g* for 6 min at 4 °C. The nuclear pellets were then incubated on ice for 15 min in buffer C (20 mM HEPES [pH 7.9], 0.42 M NaCl, 1.5 mM $MgCl_2$, 0.2 mM EDTA, 25% glycerol), after which the extract was centrifuged at 4 °C for 10 min at 26,000× g. The supernatant was then collected, divided into aliquots, and stored at −86°C. Protein concentrations were determined using the Coomassie-Plus Protein Assay Reagent (Pierce, Rockford, IL) standardized to bovine serum albumin according to the manufacturer's protocol. Nuclear extracts (5 μg) were incubated at room temperature for 15 min in 20 μL of reaction buffer containing 10 mM Tris-HCl (pH 7.5), 1 mM $MgCl_2$, 0.5 mM EDTA, 0.5 mM DTT, 50 mM NaCl, and 4% glycerol, with a ^{32}P-end-labeled, double-stranded oligonucleotide probe specific for the κB site, 5′-GCCATGGGGGGATCCCCG AAG TCC-3′ (Geneka Biotechnology Inc., Canada) and 1 μg of poly(dI-dC)•poly(dI-dC). The complexes were resolved on 5% polyacrylamide gels in Tris-HCl (pH 8.0)-borate-EDTA buffer at 10 V/cm. Dried gels were exposed using Kodak Biomax MS film (Kodak, Rochester, NY) for 1–24 h at −70 °C. Samples from the control, LPS-stimulated, and, where appropriate, hMSC-CM-treated neutrophils were all run on the same gel. Densitometry was performed using an imaging system and analysis software (BioRad, Hercules, CA).

Statistical Analysis

To limit variability in each experimental condition, each group of mice was prepared and studied at the same time. Lung injury scoring, immunostaining, ELISAs, and flow cytometry analyses were performed on separate groups of mice. The human neutrophils were collected from six patients and these samples were individually analyzed in EMSA and MMP activity assays. Data are presented as the mean ± standard error of the mean or as the mean ± standard deviation (SD) for each experimental group. One-way analysis of variance (ANOVA) and the Tukey-Kramer Multiple Comparisons test (for multiple groups) or Student's *t*-test (for two groups) were used. P-values less than 0.05 were considered statistically significant.

1. Matute-Bello G, Winn RK, Jonas M, Chi EY, Martin TR, Liles WC. Fas (cd95) induces alveolar epithelial cell apoptosis in vivo: Implications for acute pulmonary inflammation. *The American journal of pathology* 2001;158:153-161.
2. Yang KY, Arcaroli JJ, Abraham E. Early alterations in neutrophil activation are associated with outcome in acute lung injury. *Am J Respir Crit Care Med* 2003;167:1567-1574.
